# Abdominal angiostrongyliasis can be diagnosed with a immunochromatographic rapid test with recombinant galactin from *Angiostrongylus cantonensis*

**DOI:** 10.1590/0074-02760200201

**Published:** 2020-09-21

**Authors:** Carlos Graeff-Teixeira, Vanessa Fey Pascoal, Rubens Rodriguez, Alessandra Loureiro Morassutti, Pewpan M Intapan, Wanchai Maleewong

**Affiliations:** 1 Universidade do Espírito Santo, Centro de Ciências da Saúde, Departamento de Patologia e Núcleo de Doenças Infecciosas, Vitória, ES, Brasil; 2 Pontifícia Universidade Católica do Rio Grande do Sul, Escola de Ciências da Saúde, Porto Alegre, RS, Brasil; 3 Instituto de Patologia, Passo Fundo, RS, Brasil; 4 Khon Kaen University, Faculty of Medicine and Mekong Health Science Research Institute, Department of Parasitology and Excellence in Medical Innovation, and Technology Research Group, Khon Kaen, Thailand

**Keywords:** angiostrongyliasis, eosinophilic enterocolitis, eosinophilic hepatitis

## Abstract

*Angiostrongylus costaricensis* is the causative agent of abdominal angiostrongyliasis, a zoonotic infection that may produce severe eosinophilic enterocolitis or hepatitis in humans. Parasites are usually not released in stools and serology has an important role in diagnosis. Since cross-reactivity is demonstrated between *A. costaricensis* and another metastrongylid worm, *A. cantonensis*, we tested heterologous recombinant galectin as a probe in an immunochromatographic rapid diagnostic test (ICT-RDT) for detection of anti-*A. costaricensis* antibodies. Almost all (11/12) positive control sera from *A. costaricensis* infected patients were positive at ICT RDT. These are preliminary indications that r-galectin ICT-RDT is useful for diagnosing *A. costaricensis* infection.

Abdominal angiostrongyliasis (AA) is caused by *Angiostrongylus costaricensis*, an intra-arterial nematode from Mestastrongyloidea superfamily. The worms live inside mesenteric arteries in rodents, their first stage larvae (L1) are released in feces and develop into third stage larvae (L3) in fibromuscular tissues of terrestrial mollusks.^1^ L3 is infective for vertebrates and transmission occurs through ingestion, including man as an accidental host. The human infection may manifest as an eosinophilic and granulomatous enterocolitis and hepatitis.^2^ Abdominal angiostrongyliasis have been detected in most countries in Americas, from southern Unites States to northern Argentina. There are indications that most of the infections are oligosymptomatic or without any clinical manifestations.^3^ Only a few patients present with complicated acute abdomen syndromes requiring surgical treatment, especially for resection of necrotic and/or thickened intestinal segments. Since notification is not mandatory, there are no precise incidence estimates, but a reference pathological laboratory (Institute of Pathology, Passo Fundo) in the endemic area in Brazil’s southernmost State, Rio Grande do Sul (28º 15’ 46” S; 52º 24’ 24” W), have diagnosed AA approximately three times per year in the years 2014-2018 (Unpublished observations).

Another metastrongylid nematode causing human disease is *A. cantonensis*. With a biological cycle similar to *A. costaricensis*, but distinguished by the final habitat for worms in the pulmonary arteries and development of larvae in transit through cerebral tissues leading to eosinophilic meningitis.^4^ *A. cantonensis* originally came from southeastern Asian countries and Islands in the Pacific Ocean and has spread across many countries.^5^

Parasitological diagnosis is not an option in angiostrongyliasis, since larvae in feces was never consistently detected in AA patients and only rarely documented in cerebrospinal fluid in eosinophilic meningitis.^6^ Development of immunological methods have faced two main difficulties: (i) large production of antigens is laborious and it depends on *in vivo* maintenance of parasites, especially for *A. costaricensis*, which shows less than optimal adaptability to laboratory mice strains;^7^ (ii) lack of a large reference panel of sera with precise characterisation of true-positive samples by histopathology. Complicated abdominal angiostrongyliasis leading to biopsy or anatomopathological examination and identification of the parasite is not frequent.^3^ Moreover, central nervous system lesions produced by *A. cantonensis* are usually not investigated with biopsy and larvae are seldom detected in cerebrospinal fluid preventing etiological confirmation.^6^

Tissue dwelling parasites, especially those that do not complete their cycle, like angiostrongylid worms in humans, may not release any parasitic structure in body fluids or excreta. Therefore, serology stands as the main diagnostic tool. Crude worm antigens were employed in a Latex agglutination test in use in Costa Rica for decades. Eggs and small peptides were preliminarily assayed, but not applied in routine diagnosis.^8^ ^,^ ^9^

Detection of serum IgG in an immunoenzymatic assay (IgG-ELISA) employing crude female *A. costaricensis* worm antigens^10^ was standardised for diagnosis of abdominal angiostrongyliasis. Crude female antigens from *A. cantonensis* was later tested and replaced *A. costaricensis* antigens, in order to overcome the difficulties to obtain antigenic preparations.^11^ Use of heterologous antigens, i.e. cross-reacting antigens coming from filogenetically close species or from other sources, has been investigated for other helminthiasis diagnosis. One example is keyhole limpet haemocianin (KHL), a molecule sharing carbohydrate moieties cross-reacting with surface molecules on young *Schistosoma* spp. worms.^12^ Another example is *Schistosoma mansoni* antigens for diagnosing urinary schistosomiasis, caused by *S. hematobium*.^13^ Cross-reactivity between *Angiostrongylus* congeneric species have been demonstrated: anti-*A. cantonensis* antibodies recognises *A. costaricensis* antigens^14^ and vice versa, sera from *A. costaricensis* infected patients also recognise crude *A. cantonensis* antigens.^11^

Immunological diagnosis of *A. cantonensis* infection is far more advanced than with abdominal angiostrongyliasis.^5^ ^,^ ^15^ ^,^ ^16^ ^,^ ^17^ After a recent standardisation of a rapid diagnostic test (RDT) for IgG antibody detection through a immunochromatographic kit (RDT-ICT) using a recombinant galectin antigen from *A. cantonensis* ^18^ a collaboration study was established to verify the sensitivity of RDT-ICT to detect anti-*A. costaricensis* antibodies. Galectins are a class of proteins that bind to N-glycans and Galactoseβ1-4fucose is one target already identified in nematodes, including *Ascaris suum* and other helminthes of veterinary importance.^19^ Since ascariasis is a prevalent human infection in many areas, the possibility of antibody cross-reactivity between galectins from angiostrongylid and ascarid worms is to be addressed in future specificity evaluations.

A batch of 20 tests was sent from Khon Khaen University in Thailand for this preliminary evaluation with sera from individuals with confirmed (12 samples) and suspected (four samples) diagnosis of abdominal angiostrongyliasis with objective of evaluating the efficacy of r-galectin as a probe to capture anti-*A. costaricensis* antibodies. The samples were sent for routine serology and the patients gave informed consent at the time of collection for anonymously storage in the serum reference panel for angiostrongyliasis (according to Brazilian regulations and with the Helsinki Declaration of 1975, as revised in 1983; the protocol was approved by PUCRS ethics committee CEP 01/96).

Immunochromatographic RDT was performed according to manufacturers’ instructions (Kestrel BioSciences Co., Pathumthani, Thailand). Briefly, 5 µL of diluted serum samples (1:30) was filled in a sample well, followed by 90 µL of running buffer. The result was presented through visual examination within 10 min after the addition of the running buffer. If red bands appeared at both T and C lines, the result was positive and if a red band appeared only at the C line, the result was negative. The band intensity was interpreted visually according to the reference board (with 0.5 as the cut-off level). The RDT-ICT kits that revealed T line intensities greater than the cut-off point were regarded as positive results. Pooled positive and negative reference sera were produced by mixing equal volumes of Thai human serum samples from 10 eosinophilic meningitis or meningoencephalitis and clinical suspected angiostrongyliasis cantonensis patients and 10 normal healthy volunteers, respectively and were also used for testing as internal controls.

IgG-ELISA was performed as routine diagnostic method at the Molecular Parasitology Laboratory, Pontifícia Universidade Católica do Rio Grande do Sul, Porto Alegre, Brazil, with accuracy estimates of 88% sensitivity and 78% specificity.^11^ Briefly, polystyrene plates were sensitised with 0.5 µg of antigen per well and test sera were diluted at 1:200.

Except for one sample with a 0.5 degree line intensity, 11 out of 12 (91.6% sensitivity, 90.41-92.91, 95% confidence interval) positive controls serum samples from patients with identification of adult worms or eggs had positive detection of antibodies by the RDT, with an even distribution of intensities from 1.0 to 4.0 (Table). Reactivity of these same sera was tested by IgG-ELISA with ratios of positivity from 1.07 to 51.5 and a very low coefficient of correlation: 0.19288. The only positive control serum had an ELISA ratio of 11.5. It is important to note that ELISA employs a very complex set of proteins while RDT-ICT has a unique recombinant protein, what may explain lack of correlation between ELISA ratios and intensity of bands in RDT-ICT. From four samples without identification of parasitic structures in histopathological examination, but with a suspected diagnosis based on histopathological criteria, three were RDT-ICT positive with degrees of intensity: one, two and three.

**TABLE:** Ratio values for anti-*Angiostrongylus costaricensis* IgG enzyme-linked immunosorbent assay (ELISA), band intensities obtained in a recombinant galectin immunochromatographic (ICT) rapid diagnostic test (RDT) and histopathological findings defining 12 true-positive samples and four suspected abdominal angiostrongyliasis samples from southern Brazil, 2014 to 2018. ICT-RDT is considered positive with band intensities higher than 0.5

IgG-ELISA ratio	ICT-RDT Band intensity	ICT-RDT result	Histopathology findings
True-positive samples			
11.55	0.5	negative	worms
5.62	1	positive	worms
1.22	1	positive	worms
51.5	2	positive	worms
17.4	2	positive	eggs
4.74	2	positive	worms
3.4	3	positive	worms
2.94	3	positive	worms
1.3	3	positive	worms
15	4	positive	worms
19.05	4	positive	eggs
1.07	4	positive	eggs
Suspected abdominal angiostrongyliasis			
4.58	0	negative	eosinophilia
1.43	1	positive	eosinophilia
2.52	2	positive	eosinophilia
1.07	3	positive	necrosis

In conclusion, these are preliminary indications that RDT-ICT with heterologous r-galectin from *A. cantonensis* is useful for serological diagnosis of abdominal angiostrongyliasis in individuals presenting suggestive clinical and laboratorial findings. While *A. cantonensis* infections manifest as meningitis, *A. costaricensis* causes intestinal or hepatic pathology, what allows the differential etiological diagnosis in clinical settings. In areas with occurrence of both parasites, serology is not an adequate tool for prevalence studies, because of the extensive cross-reactivity already mentioned.^11^ ^,^ ^14^ A prospective multicentric longitudinal study of accuracy of RDT-ICT in *A. costaricensis* endemic areas, including a well-characterised negative control panel for specificity evaluation is needed. The new diagnostic tool shall contribute both to epidemiological surveys and better management of patients with abdominal angiostrongyliasis.
